# Blood pressure is elevated in the absence of resistance artery dysfunction in a mouse model of diet-induced obesity

**DOI:** 10.3389/fphys.2025.1602155

**Published:** 2025-08-05

**Authors:** Darcy Lidington, Danny D. Dinh, Nan Chen, Hangjun Zhang, Yu-Qing Zhou, Scott P. Heximer, Daniel A. Winer, Alexandre Martchenko, Steffen-Sebastian Bolz

**Affiliations:** ^1^Department of Physiology, University of Toronto, Toronto, ON, Canada; ^2^The Ted Rogers Centre for Heart Research, Translational Biology and Engineering Program, University of Toronto, Toronto, ON, Canada; ^3^ Qanatpharma Ltd., Toronto, ON, Canada; ^4^Division of Cellular and Molecular Biology, Diabetes Research Group, Toronto General Hospital Research Institute (TGHRI), University Health Network, Toronto, ON, Canada; ^5^Department of Laboratory Medicine and Pathobiology, University of Toronto, Toronto, ON, Canada; ^6^ Buck Institute for Research on Aging, Novato, CA, United States; ^7^Department of Immunology, University of Toronto, Toronto, ON, Canada; ^8^ Qanatpharma AG, Stans, Switzerland; ^9^ Aphaia Pharma AG, Zug, Switzerland

**Keywords:** obesity, insulin resistance, myogenic activity, resistance arteries, echocardiography, impaired glucose tolerance (IGT), hypertension, vascular reactivity

## Abstract

**Introduction:**

Hypertension and impaired tissue perfusion are frequent comorbidities in obesity. Since resistance arteries are the primary regulators of peripheral resistance and hence, systemic blood pressure and local blood flow control, we hypothesized that resistance arteries isolated from obese mice would display augmented myogenic reactivity and altered vasomotor responses, compared to non-obese controls.

**Methods:**

Eight-week-old C57BL/6J mice were fed either a high-fat diet (60% calories from fat; HFD) or a matched control diet for 16 weeks. Body weight, fasting blood glucose, oral glucose tolerance and insulin tolerance were measured. In parallel studies, we measured mean arterial pressure, conducted echocardiographic measurements of cardiac morphology and function and assessed skeletal muscle, mesenteric and cerebral resistance artery reactivity *ex vivo* with pressure myography.

**Results:**

HFD mice exhibited substantial weight gain and metabolic dysfunction compared to controls. Left ventricular wall thickness and mass were increased in HFD mice, but no other morphological or functional cardiac parameters were different from controls. Blood pressure was modestly increased in HFD mice (from 81 to 87 mmHg; measured under anesthesia); however, contrary to our hypothesis, resistance arteries from HFD mice showed no overt microvascular phenotype in any microvascular bed tested (i.e., no differences in passive diameter, myogenic reactivity or vasomotor responses to phenylephrine or acetylcholine).

**Conclusion:**

We conclude that resistance artery function is unaltered in this diet-induced model of obesity with metabolic dysfunction.

## Introduction

Obesity is a global scale public health concern. According to World Health Organization statistics, worldwide adult obesity has more than doubled since 1990 and currently, 1 in 8 individuals are classified as obese (World Health Organization, 2019; Menifield et al., 2008). Obesity frequently induces a pre-diabetic status termed “metabolic syndrome,” a state of systemic inflammation that underpins the development of established risk factors for cardiovascular disease, stroke and type 2 diabetes (Engin, 2017; Franssen et al., 2011; Vykoukal and Davies, 2011; Dutta et al., 2024). Clinically, obesity is defined as a body mass index (BMI; weight/height^2^) of over 30 kg/m^2^; metabolic syndrome is present if three or more of the following five criteria are met: 1) waist circumference over 102 cm in males or 88 cm in females; 2) blood pressure over 130/85 mmHg; 3) fasting triglyceride level over 150 mg/dL; 4) fasting high-density lipoprotein (HDL) cholesterol level less than 40 mg/dL (men) or 50 mg/dL (women); and 5) and fasting blood sugar over 100 mg/dL (Stone et al., 2005).

Microvascular dysfunction is a well-known component of metabolic syndrome, which manifests as impaired tissue blood flow control (Frisbee and Delp, 2006; Livingston et al., 2020; Stepp et al., 2004; Carter et al., 1985) and/or hypertension (Leggio et al., 2017; Garr et al., 1987). The arterial vascular tree can be broadly separated into macro- and micro-vascular components, each with distinct functional characteristics: macrovessels (conduit arteries) are large, compliant arteries that conduct blood with minimal resistance, while microvessels (resistance arteries and arterioles) generate the majority of vascular resistance in the cardiovascular system and are instrumental in controlling blood flow and organ perfusion (Davis et al., 1986; Chantler and Frisbee, 2015). In addition to playing a prominent role in the regulation of peripheral resistance and organ perfusion (Butcher et al., 2013), resistance arteries are also tasked with protecting smaller arterioles and capillaries from high pressures (Konecny et al., 1985). It is not surprising, therefore, that perturbing resistance artery function can have wide-ranging effects, most notably malperfusion and increased tissue injury susceptibility (Osmond et al., 2009). Myogenic reactivity, the ability to dynamically match vascular resistance to luminal pressure (Lidington et al., 2013), is a hallmark feature of resistance arteries and an ideal readout to mechanistically link resistance artery function (and dysfunction in pathological settings) to organ perfusion and systemic hemodynamic parameters (Lidington et al., 2019; Kroetsch et al., 2017).

Surprisingly, few studies use mouse models to assess the effect of diet-induced obesity on myogenic reactivity and these reports are limited to mesenteric arteries (Dunn et al., 2017; Ogalla et al., 2015; Krüger et al., 2020). The present study targeted this deficit by assessing the effect of a standard high-fat diet model on resistance arteries from 5 vascular beds, including 2 from skeletal muscle, 2 from the cerebral microcirculation and mesenteric arteries. Studies in rat models suggest that myogenic reactivity may be broadly, but not uniformly, altered by obesity: enhanced myogenic vasoconstriction has been observed in skeletal muscle and cerebral resistance arteries (Butcher et al., 2013), while attenuated reactivity has been reported in mesenteric resistance arteries (Sweazea and Walker, 2012). We therefore hypothesized that high-fat diet induced obesity would alter resistance artery myogenic reactivity in all vascular beds tested, but not necessarily in the same manner.

## Methods

This investigation conforms to the National Research Council’s 2011 Guide for the Care and Use of Laboratory Animals (ISBN: 0-309-15400-6). All experimental procedures were approved by the Institutional Animal Care and Use Committee at the University of Toronto (Protocol ID# 20013119) and the Animal Care Committee at the University Health Network, Toronto (Protocol ID# 2570).

### Mice

Wild-type male C57BL/6J mice (RRID: IMSR_JAX:000664) were purchased from The Jackson Laboratory (Bar Harbor, United States) at 6 weeks of age. The mice were housed in a controlled climate (21^o^C, 40%-60% humidity) with a standard 12h:12h light-dark cycle and access to water and food *ad libitum*. Following 2 weeks acclimatization, the mice were placed on either a high-fat diet (HFD; 60% calories from fat; Research Diets Inc., New Brunswick, United States; cat# D12492) or a matched control chow (NC; 10% calories from fat; Research Diets Inc.; cat# D12450J) with *ad libitum* access to water and food. Endpoints were assessed after 16–20 weeks on the HFD/NC diets. All parameter assessments were conducted during the normal workday and coincided with the lights-on phase in the light-dark cycle.

### Isolation and functional assessment of resistance arteries

Cremaster skeletal muscle (Kroetsch et al., 2017), radial skeletal muscle (Phan et al., 2022) and mesenteric (Sauve et al., 2016) resistance arteries were dissected, cannulated onto micropipettes, stretched to their *in vivo* lengths, and pressurized to 60 mmHg. Posterior and olfactory cerebral arteries were dissected, cannulated and pressurized to 45 mmHg (Yagi et al., 2015; Yang et al., 2012). For myographical assessments, arteries were imaged with a CCD camera at ×20 (mesenteric) or ×40 (all others) magnification. Luminal diameter was measured using a Crescent Electronics (Windsor, Canada) video edge detector and logged using Photon Technology International FeliX32 analysis software (Horiba Canada Inc.; London, Canada). Myography experiments were conducted in calcium-containing 3-morpholinopropanesulfonic acid (MOPS) buffered saline at 37^o^C with no perfusion ([mmol/L]: NaCl 147.0, KCl 4.7, CaCl_2_ 1.5, MgSO_4_ 1.2, NaH_2_PO_4_ 1.2, pyruvate 2.0, EDTA 0.02, MOPS 3.0 and glucose 5.0). Vessels with leaks due to improper cannulation or small branches (evident as flow-related disturbances/movements upon pressurization) were considered compromised and excluded. Vasomotor responses to 10 μmol/L phenylephrine (*Millipore Sigma*; Oakville, Canada; cat# P6126) provided an assessment of vessel viability at the start of each experiment: skeletal and mesenteric arteries failing to show ≥50% constriction in response to phenylephrine were considered damaged/compromised and excluded; cerebral arteries failing to show ≥30% constriction were excluded. Viable arteries were presumed to have an intact endothelium, and no manipulations were undertaken to denude them.

Myogenic responses were elicited by stepwise 20 mmHg increases in transmural pressure, which was manipulated by changing the height of a MOPS buffer-filled fluid column. At each pressure step, vessel diameter (dia_active_) was measured once a steady state was reached. Following completion of all dia_active_ measurements, the MOPS buffer was replaced with a Ca^2+^-free version and maximal passive diameter (dia_max_) was recorded at each pressure step. We used a 20–100 mmHg pressure ramp for radial and cremaster skeletal muscle, mesenteric and posterior cerebral arteries and a 20–80 mmHg pressure ramp for olfactory cerebral arteries. We used a lower top pressure for olfactory arteries, as we found that the 100 mmHg pressure level damaged the artery and compromised its function for subsequent measures (unpublished observations).

Myogenic tone was calculated as the percent constriction in relation to the maximal diameter at each respective transmural pressure: tone (% of dia_max_) = [(dia_max_-dia_active_)/dia_max_]x100, where dia_active_ is the vessel diameter in MOPS containing Ca^2+^ and dia_max_ is the diameter in Ca^2+^-free MOPS. Analyses of vasomotor responses to phenylephrine, sodium nitroprusside (*Millipore Sigma*; cat# A6625) and acetylcholine (*Millipore Sigma*; cat# 71778) used the same calculation, only in this case, dia_active_ represents the vessel diameter at steady state following application of the given agent. Responses to acetylcholine and sodium nitroprusside were determined in resistance arteries pre-constricted with 3 μmol/L phenylephrine.

### Echocardiography and blood pressure measurements

Echocardiographic measurements (using M-mode, B-mode imaging of the left ventricle and pulsed Doppler velocity-time integral (VTI) at the aortic orifice) were performed under anesthesia and collected using a high frequency ultrasound system with a 30 MHz linear array transducer (Vevo 3100; Fujifilm VisualSonics, Toronto, Canada). Cardiac output (CO) was calculated using VTI measures and aortic diameter. Left ventricular mass was calculated using M-mode measurements of the left ventricular internal diameters, anterior and posterior wall thicknesses, based on the Devereux formula (Devereux et al., 1986). We conducted mean arterial pressure measurements under anesthesia immediately following the echocardiographic assessments, using a Millar SPR-671 micro-tip mouse pressure catheter (Inter V Medical Inc., Montreal, Canada). Total peripheral resistance (TPR) was calculated as TPR = MAP/CO.

### Blood collection and metabolic measurements

Oral glucose and intraperitoneal insulin tolerance tests were conducted on 6 h fasted mice. A basal blood measurement was performed at t = 0 min, followed by either an oral glucose tolerance test (OGTT; gavage 2 g glucose/kg body weight) or an insulin tolerance test (0.75U/kg body weight i.p.). Glucose measurements for each test were taken at t = 15, 30, 60, 90 and 120 min. Blood glucose was determined using a OneTouch blood glucometer (LifeScan; Malvern, United States). Area under the curve (AUC) was calculated by trapezoidal interpolation with GraphPad Prism 9 software (San Diego, United States), using baseline-corrected data (i.e., change in blood glucose).

### Data collection and statistics

58 mice were used in the present study; only male mice were utilized. Upon arrival to the animal facility, mice were distributed into cages without a randomization methodology and acclimatized. Acclimatized animal cages were randomly assigned to either the HFD or NC using a web-based randomizer (http://www.randomizer.org). It was not possible to collect data under blinded conditions, due to the obvious size/weight difference betweeen the HFD/NC groups.

**FIGURE 1:**
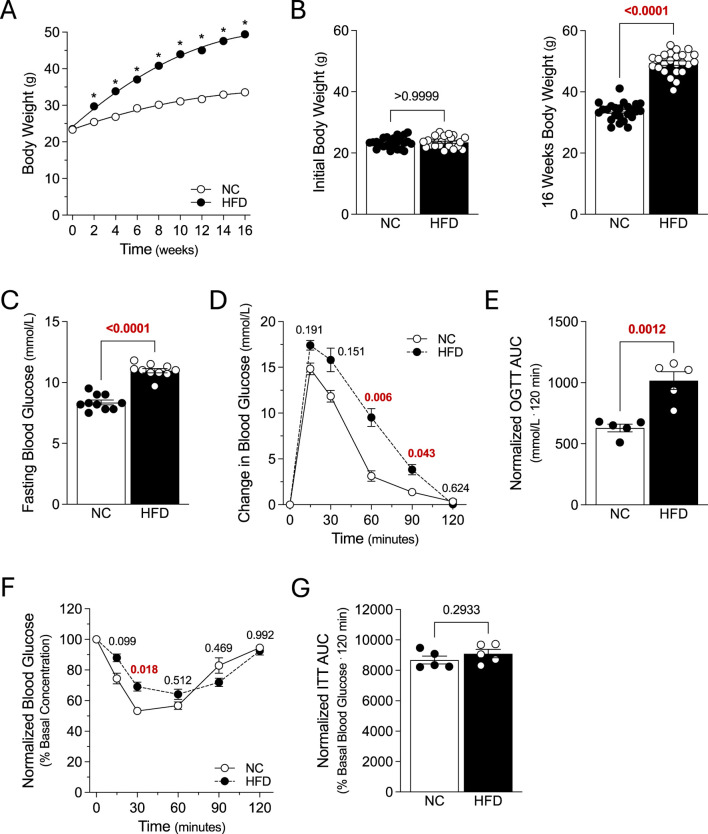
Metabolic Characteristics. Mice were fed either a normal control diet (NC) or high-fat diet (HFD) for 16 weeks, with body weight measured every 2 weeks. **(A)** displays average bodyweight over time for the NC and HFD groups (both N = 24 mice). **(B)** NC and HFD mice (both groups N = 24 mice) had similar weights at study start (*left panel*); after 16 weeks, HFD mice gained significantly more weight than NC controls (right panel). **(C)** Fasting blood glucose was significantly higher in HFD mice, compared to NC controls (both groups N = 10 mice). **(D)** Plotted are blood glucose measures following an oral glucose tolerance test (OGTT) in 6 h fasted mice (2 g/kg; both groups N = 5 mice). **(E)** Area under the curve measures (AUC) confirm that oral glucose tolerance is significantly compromised in HFD mice, compared to NC controls (both N = 5 mice). **(F)** Plotted are blood glucose measures following an *i.p.* injection of insulin (0.75U/kg) in 6 h fasted mice (both groups N = 5 mice). **(G)** AUC measures for the insulin tolerance test (ITT) in *Panel F* are not significantly different (both groups N = 5 mice). **(A, D, F)** were analyzed with a two-way ANOVA, followed by a Sídak’s multiple comparison post-test for each NC/HFD comparison. All other data were compared with an unpaired *t*-test. In **(A)**, * denotes a significant difference (P < 0.05) for the NC/HFD comparison; note that the error bars or too small for visualization. *Panel B* displays the expanded data for study start and after 16 weeks and the Sídak’s multiple comparisons post-test results.

**FIGURE 2:**
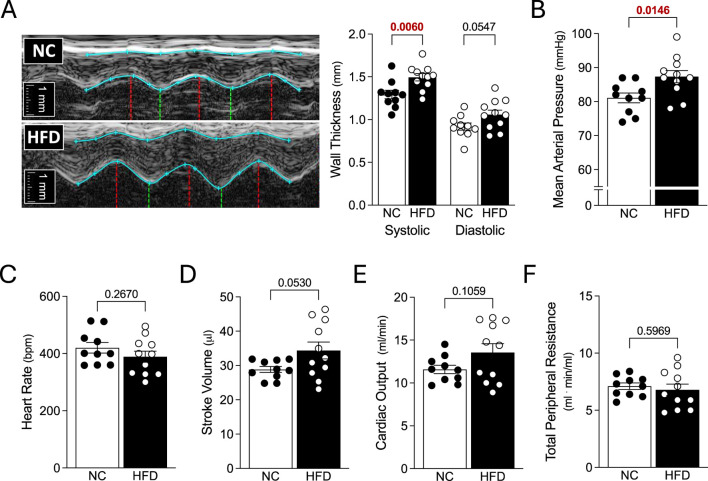
Echocardiography and Systemic Hemodynamics. Mice fed either a normal control diet (NC) or high-fat diet (HFD) for 16 weeks were assessed with echocardiography and invasive blood pressure measurements. **(A)** Shown are representative M-mode echocardiographic images of the left ventricular anterior wall. The blue lines show the wall outlines (i.e., the epicardium and endocardium); the red vertical lines indicate the end-diastole and the green vertical lines indicate the peak-systole. To the right of the images are the compiled measures of wall thicknesses at peak-systole and end-diastole. **(B)** HFD mice had higher mean arterial pressure than NC mice. However, **(C)** heart rate, **(D)** stroke volume **(E)** cardiac output and **(F)** total peripheral resistance were not statistically different between the NC and HFD groups. Stroke volume and cardiac output **(D,E)** were compared with a Welch’s corrected unpaired *t*-test; all other data were compared with an unpaired *t*-test. For all data, N = 10 mice for NC and N = 11 mice for HFD.

**FIGURE 3:**
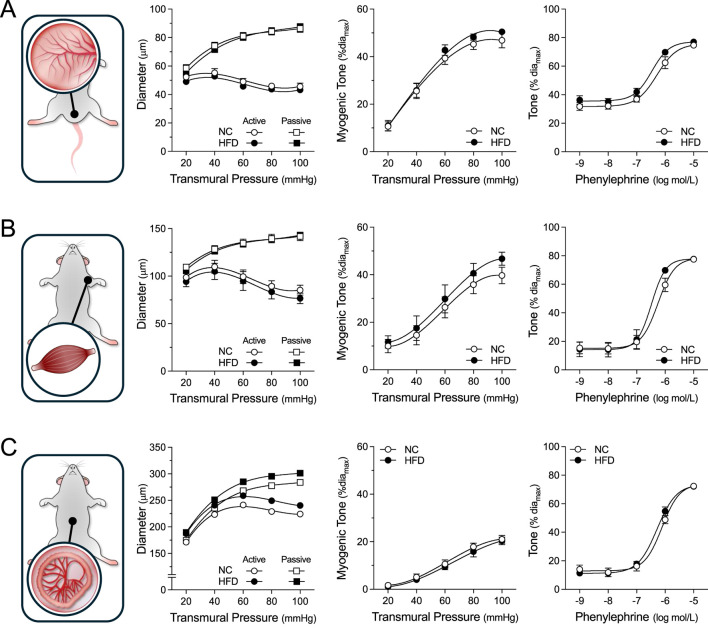
Vascular Reactivity in Peripheral Resistance Arteries. Shown are measured active and passive diameters (*left*), the corresponding myogenic tone calculations (*center*) and phenylephrine dose-response relationships (*right*) for cremaster skeletal muscle, forearm radial skeletal muscle and mesenteric resistance arteries isolated from mice fed either a normal control diet (NC) or high-fat diet (HFD) for 16 weeks. **(A)** In cremaster skeletal muscle resistance arteries (both groups n = 8 vessels from N = 4 mice), no NC/HFD differences are observed in passive/active diameters, myogenic tone or phenylephrine-stimulated vasoconstriction. **(B)** In radial skeletal muscle resistance arteries (NC n = 8 vessels from N = 4 mice; HFD n = 7 vessels from N = 4 mice), no NC/HFD differences are observed in passive/active diameters, myogenic tone or phenylephrine-stimulated vasoconstriction. **(C)** In mesenteric resistance arteries (both group n = 6 vessels from N = 3 mice), no NC/HFD differences are observed in passive/active diameters, myogenic tone or phenylephrine-stimulated vasoconstriction. All data are compared with a two-way ANOVA; no significant differences were observed between the NC and HFD groups.

**FIGURE 4:**
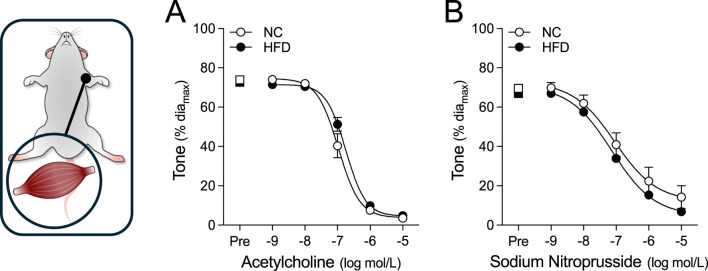
Vasodilator responses in radial skeletal muscle resistance arteries. Radial skeletal muscle resistance arteries were isolated from mice fed either a normal control diet (NC) or high-fat diet (HFD) for 16 weeks (all groups n = 6 vessels from N = 3 mice). Arteries were pre-constricted (Pre) with 3 μmol/L phenylephrine and co-treated with increasing concentrations of **(A)** acetylcholine or **(B)** sodium nitroprusside. Radial arteries dilate to both acetylcholine and sodium nitroprusside. All data are compared with a two-way ANOVA; no significant differences were observed between the NC and HFD groups.

**FIGURE 5:**
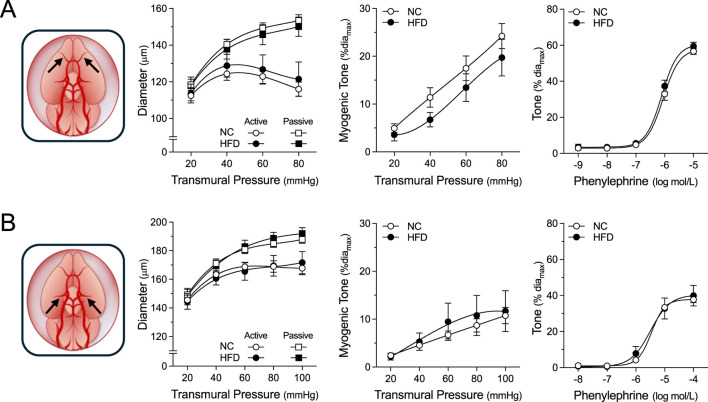
Vascular Reactivity in Cerebral Resistance Arteries. Shown are measured active and passive diameters (*left*), the corresponding myogenic tone calculations (*center*) and phenylephrine dose-response relationships (*right*) for olfactory and posterior cerebral resistance arteries isolated from mice fed either a normal control diet (NC) or high-fat diet (HFD) for 16 weeks. **(A)** In olfactory cerebral resistance arteries (NC n = 11 vessels from N = 6 mice; HFD n = 8 vessels from N = 4 mice), no NC/HFD differences are observed in passive/active diameters, myogenic tone or phenylephrine-stimulated vasoconstriction. **(B)** In posterior cerebral resistance arteries (NC n = 9 vessels from N = 5 mice; HFD n = 6 vessels from N = 4 mice), no NC/HFD differences are observed in passive/active diameters, myogenic tone or phenylephrine-stimulated vasoconstriction. All data are compared with a two-way ANOVA; no significant differences were observed between the NC and HFD groups.

All data are expressed as means ± standard error of the mean (SEM). In Figures 1, 2, N refers to the number of mice. Myography experiments attempted to collect data from two vessels per mouse, but in some cases, vessels were excluded as non-viable: thus, in Figures 3–5, n refers to the numbers of vessels and N refers to the number of mice. Data were statistically analyzed using Graphpad Prism 9 software. Prior to conducting statistical comparisons, we assessed data normality (Shapiro-Wilk test) and variance homogeneity (F test). Weight gain (over time), oral glucose tolerance, insulin tolerance and myographic measurements (i.e., myogenic tone and vasomotor responses) were analyzed with a Greenhouse-Geisser corrected two-way ANOVA with paired comparisons for time/pressure/concentration and unpaired comparisons for the NC/HFD groups. We did not use post-tests to compare the repeated measures, as a positive ANOVA result was sufficient to confirm a response (all ANOVA analyses were significant for paired comparisons); we used a Sídak’s multiple comparisons post-test for NC/HFD comparisons, when the ANOVA was significant for the group NC/HFD effect. For all single group comparisons, normal data with equal variances were analysed with a two-tailed independent *t-*test; normal data with unequal variances were analysed with a two-tailed Welch’s *t-*test; non-normal data were analysed with a two-tailed Mann-Whitney U Test. Differences were considered significant at P < 0.05.

## Results

### Metabolic characteristics of high fat diet model

Mice (8 weeks of age) were fed either a NC diet or HFD for 16 weeks, with body weight measured every 2 weeks. The two groups had similar weights at study start; as expected, the HFD group rapidly diverged from the NC group and was significantly heavier from the 2-week timepoint onwards (Figures 1A,B). At 16 weeks post-diet, HFD mice had increased: 1) fasting blood glucose (Figure 1C); 2) glucose levels at 60 and 90 min post-OGTT (Figure 1D); and 3) OGTT area under the curve (Figure 1E). HFD mice also displayed elevated glucose levels at 30 min post-insulin injection (Figure 1F); however, the insulin tolerance area under the curve was not different compared to NC controls (Figure 1G). Collectively, these results confirm the successful generation of obese mice with a metabolic phenotype.

### Echocardiography and Systemic Hemodynamics

HFD mice exhibited significant increases in systolic left ventricular anterior wall thickness and left ventricular mass; diastolic left ventricular anterior wall thickness and posterior wall thickness were not statistically different than controls (Table 1). Cardiac function was not compromised: systolic and diastolic ventricular diameters/areas, fractional shortening, fractional area change, stroke volume, heart rate and cardiac output were all not statistically different compared to NC controls (Figure 2 and Table 1). We observed a significant increase in mean arterial pressure; total peripheral resistance, however, was not different (Figure 2). Collectively, these *in vivo* data support the conclusions that: 1) cardiac function is not compromised in HFD mice, although there is evidence for the initial stages of ventricular remodelling; and 2) systemic vascular resistance is not elevated in HFD mice, although a modest blood pressure phenotype is present.

**TABLE 1:** Echocardiographic measurements.

Parameter	NC	HFD	Test	P-Value
Systolic LVAW Thickness (mm)	1.29 ± 0.05	1.50 ± 0.04	t-test	0.006
Diastolic LVAW Thickness (mm)	0.92 ± 0.04	1.06 ± 0.05	t-test	0.055
Systolic LVPW Thickness (mm)	1.15 ± 0.04	1.23 ± 0.04	t-test	0.213
Diastolic LVPW Thickness (mm)	0.81 ± 0.03	0.88 ± 0.02	t-test	0.082
Left Ventricular Mass (mg)	133.8 ± 4.8	156.6 ± 8.2	t-test	0.030
Systolic Diameter (mm)	2.80 ± 0.08	2.68 ± 0.11	t-test	0.401
Diastolic Diameter (mm)	4.08 ± 0.10	4.04 ± 0.13	Mann-Whitney	0.654
Fractional Shortening (%)	31 ± 1	34 ± 1	t-test	0.171
Systolic Left Ventricular Area (mm2)	15.5 ± 0.5	14.4 ± 1.0	Welch's t-test	0.320
Diastolic Left Ventricular Area (mm2)	23.8 ± 0.6	23.2 ± 1.3	Welch's t-test	0.679
Fractional Area Change (%)	35 ± 1	38 ± 2	t-test	0.213
Stroke Volume (*μ*l)	28.8 ± 0.9	34.4 ± 2.4	Welch's t-test	0.053
Heart Rate (beats/minute)	404 ± 19	395 ± 14	Mann-Whitney	0.546
Cardiac Output (ml/minute)	11.6 ± 0.5	13.6 ± 1.0	Welch's t-test	0.104

Mice were fed either a normal control diet (NC) or high-fat diet (HFD) for 16 weeks prior to echocardiographic measurements. Data are means ± standard error measurements, with N = 11 mice for NC and n = 10 mice for HFD. The statistical test used for group comparison is listed in the column entitled “Test”.

### Myography studies

For all myographic assessments (i.e., active/passive diameter, myogenic tone and vasomotor tone), the repeated measures component of the two-way ANOVA statistical test confirmed that pressure or concentration had a significant effect on diameter/tone. Figure 3 displays functional assessments for cremaster skeletal muscle, radial skeletal muscle and mesenteric arteries. No significant HFD/NC comparison differences in active/passive diameters, myogenic tone or phenylephrine dose-response curves were observed, as assessed by two-way ANOVA. Calculated log EC_50_ values for the dose-response relationships are presented in Table 2. Interestingly, radial arteries from HFD mice displayed a small leftward shift in its phenylephrine log EC_50_ value (approximately 0.2 log units); none of the other HFD/NC comparisons for phenylephrine log EC_50_ were statistically different (Table 2). The altered log EC_50_ result in radial arteries prompted us to assess vasodilator responses in this artery type. Figure 4 displays functional data for radial artery vasodilator responses to acetylcholine and sodium nitroprusside. There was no significant HFD/NC difference in either curve, as assessed by two-way ANOVA; HFD/NC comparisons of the acetylcholine and sodium nitroprusside log EC_50_ values did not identify any significant differences (Table 2). For the radial arteries assessed in Figure 4, mean passive diameters at 60 mmHg were not statistically different between the two groups (NC = 140 ± 8 μm, n = 6 vessels from N = 3 mice; HFD = 141 ± 3 μm, n = 6 vessels from N = 3 mice; unpaired *t*-test P = 0.953). Figure 5 displays functional assessments for olfactory and posterior cerebral arteries. As for the peripheral arteries, no significant HFD/NC differences in active/passive diameters, myogenic tone or phenylephrine dose-response curves were observed, as assessed by two-way ANOVA (Figure 5); HFD/NC comparisons of the phenylephrine log EC_50_ values did not identify any significant differences (Table 2). Collectively, our *ex vivo* myography data indicate that HFD did not meaningfully alter vascular reactivity in olfactory, posterior cerebral, mesenteric or cremaster resistance arteries. HFD may have a minimal-to-small effect on phenylephrine responses in radial skeletal muscle resistance arteries, but no other assessments were altered in this vascular bed.

**TABLE 2:** Vasomotor Response log EC_50_ Values.

Vessel type	Agent	log EC50 NC	n	log EC50 HFD	n	Test	P-Value
Cremaster Skeletal Muscle	Phenylerphrine	−6.22 ± 0.14	8	−6.45 ± 0.06	8	Mann-Whitney	0.195
Radial Skeletal Muscle	Phenylerphrine	−6.22 ± 0.08	8	−6.48 ± 0.04	7	t-test	0.021
Mesenteric	Phenylerphrine	−6.08 ± 0.07	6	−6.26 ± 0.06	6	t-test	0.069
Olfactory Cerebral	Phenylerphrine	−5.95 ± 0.09	11	−6.03 ± 0.11	8	Mann-Whitney	0.717
Posterior Cerebral	Phenylerphrine	−5.44 ± 0.04	9	−5.41 ± 0.13	6	Mann-Whitney	0.689
Radial Skeletal Muscle	Acetylcholine	−6.95 ± 0.14	6	−6.70 ± 0.08	6	t-test	0.143
Radial Skeletal Muscle	Nitroprusside	−6.99 ± 0.13	6	−7.12 ± 0.02	6	Welch’s t-test	0.353

Mice were fed either a normal control diet (NC) or high-fat diet (HFD) for 16 weeks prior vasomotor response assessment. Data are means ± standard error measurements; n refers to the number of vessels assessed. The statistical test used for group comparison is listed in the column entitled “Test”.

## Discussion

Using a well-established C57BL/6J mouse model of obesity with metabolic dysfunction, this investigation assessed cardiac morphology, systemic hemodynamic parameters and vascular reactivity in isolated resistance arteries. Although we expected to observe a vascular phenotype in obese mice, the HFD model had no effects on peripheral resistance *in vivo* and a minimal effect on microvascular function *ex vivo*.

There are several monogenetic and polygenetic experimental models of obesity: some notable examples include leptin-deficient mice (*ob/ob*), leptin-receptor deficient mice (*db/db*) and the New Zealand obese mouse strain (NZO) (Martins et al., 2022). While genetics undoubtedly contribute to the risk of becoming obese, genetics alone cannot explain the sudden onset of the world-wide obesity epidemic. Indeed, human obesity is primarily linked to dietary habits and thus, diet-induced mouse models of obesity are generally considered to be a more clinically relevant than genetic models. One significant challenge when initiating a diet-induced obesity study is selecting an appropriate obesogenic diet, as there are many to choose from. This reflects a generally accepted premise that the individual dietary components can significantly impact the resulting metabolic phenotype (Buettner et al., 2007). Many studies utilize defined diets from commercial sources (e.g., the 60% high-fat diet used in the present study); however, there are also many custom obesogenic diets that contain different fats (lard, palmitic acid, vegetable oils), sugars (glucose, fructose, sucrose), salt content and even vary in palatability (Martins et al., 2022). Feeding protocols can also differ significantly (i.e., *ad libitum versus* time-restricted feeding), adding an additional layer of complexity (Hatori et al., 2012). These variations can considerably hamper the comparison of different studies, even when the core scientific questions are similar.

Not all mouse strains respond similarly to consuming a high-fat diet: some strains are considered obesity-prone (e.g., C57BL/6J, 129X1/SvJ and UM-HET3), while others are obesity-resistant (e.g., BALB/cJ and SWR/J) (Buckley et al., 2021; Zheng et al., 2023). The C57BL/6J inbred mouse is commonly used as a diet-induced obesity model, because this strain 1) remains relatively lean and without metabolic abnormalities when fed a normal chow diet *ad libitum*; 2) rapidly develops obesity, hyperinsulinemia and hyperglycemia on a high-fat diet; and 3) displays metabolic abnormalities that parallel human progression in obesity (Martins et al., 2022; Surwit et al., 1988).

As expected, we successfully generated obese mice with metabolic dysfunction in the present study. In terms of cardiac morphology, most morphologic parameters were unaltered in HFD mice, although the increased left ventricular wall thickness suggests that these mice are in the initial stages of cardiac remodelling. Cardiac function and most systemic hemodynamic parameters were unaffected in HFD mice. Thus, we conclude that the HFD model has a relatively normal cardiac phenotype after 16 weeks of obesogenic diet. Given that cardiac remodelling may be in its initial stages, it is tempting to speculate that these mice may develop a more severe pathological cardiac phenotype at later time points on the high-fat diet. We observed a significant increase in mean arterial pressure that reasonably emulates observations made in a separate study using a similar mouse model of obesity (45% HFD in C56BL/6J mice) (Asirvatham-Jeyaraj et al., 2016). However, this blood pressure increase is small from a clinical perspective (∼6 mmHg) and thus, this phenotype must be considered modest/mild.

As reviewed by Alpert et al., in humans, increased total and central blood volume is a key clinical feature of obesity that drives several hemodynamic alterations (Alpert et al., 2016). The additional blood volume increases cardiac output, almost exclusively by augmenting stroke volume; in turn, this drives a hypertensive state, frequently in the absence of peripheral resistance elevation (Alpert et al., 2016). The most common clinical cardiac morphology alteration in obesity is left ventricular hypertrophy, evident as increased left ventricular wall thickness and mass (Alpert et al., 2016). In this regard, the HFD mouse model used in the present study recapitulated several aspects of clinical obesity: specifically, we observed left ventricular wall thickening, increased left ventricular mass and elevated blood pressure in the absence of increased peripheral resistance.

Since mean arterial pressure is the product cardiac output and total peripheral resistance, we expected that one or both underlying parameters would be increased by the high-fat diet; yet, neither were statistically different compared to NC controls. We speculate that cardiac output is driving the difference in MAP, because it is the only parameter of the two with a larger mean value in HFD mice. Based on the data at hand, we were underpowered to reliably detect a significant difference in cardiac output and a sample size calculation (with 1−β = 0.80) determines that N = 24 mice per group would have been required, assuming that the effect size and variability remain similar with additional measurements.

Our *ex vivo* assessments of vascular function did not identify an effect of obesity on myogenic reactivity, vasomotor responses or passive diameter, which aligns with the lack of effect on peripheral resistance *in vivo*. In radial arteries, we observed a small leftward shift in the phenylephrine log EC_50_ value, which is surprising, given the lack of significant effect on tone at any given phenylephrine concentration. The most likely explanation is that non-significant differences in tone during the phenylephrine ramp alter the best-fit sigmoidal dose-response curve, leading to differences in log EC_50_ values. It is difficult to ascribe a physiological significance to this log EC_50_ shift: the data imply that radial arteries may be slightly more sensitive to endogenous α1-adrenergic receptor ligands, such as adrenaline and noradrenaline; however, since total peripheral resistance is not changed, it appears as though a combination of small effect size (i.e., small log EC_50_ shift) and/or limited prevalence within the vascular system (i.e., perhaps only radial resistance arteries experience this increased sensitivity) minimize its physiological relevance *in vivo*.

Our study indicates that the early stages of metabolic syndrome does not immediately elicit vascular dysfunction. This suggests that the vascular system is resilient and requires other “hits”, such as persistent/severe metabolic dysfunction, aging and hypertension. This conclusion is buttressed by a study in ApoE^−/−^ mice that compared young (6 weeks) and old (7.5 months) mice with hypercholesterolemia: young mice displayed preserved blood pressure, perfusion responses and minimal atherosclerosis, while older mice displayed elevated blood pressure, attenuated perfusion responses and atherosclerosis (Yang et al., 1999). Intriguingly, several vascular features of obesity, including intima/media thickening (Ciccone et al., 2001) and vascular rarefaction (Gavin et al., 2005) are known indices of vascular ageing (Juonala et al., 2008; Grunewald et al., 2021). Thus, it is tempting to speculate that obesity and/or metabolic dysfunction accelerates vascular aging. In the human context, this suggests: 1) that there is a window of opportunity to intervene prior to the development vascular dysfunction; 2) there should be no delay in such interventions, even if vascular function is currently unaffected; and 3) if vascular dysfunction is an “ageing” effect, may be very difficult to reverse, once it emerges.

Our study has several limitations that require acknowledgement. First and foremost, our study was limited to male mice. Sex differences in the epidemiology/pathophysiology of metabolic syndrome (Rochlani et al., 2015); energy metabolism (i.e., lipid and glucose metabolism) (Varlamov et al., 2014) and cardiovascular disease risk (Cignarella et al., 2024) are clearly evident in humans. Similarly, sex differences in visceral adiposity and metabolic dysfunction have been observed by others in diet-induced obesity mouse models (Zheng et al., 2023). At the vascular level, our previous work shows that male and female olfactory cerebral arteries possess distinctly different phenotypes, underpinned by the role the cystic fibrosis transmembrane conductance regulator (CFTR) plays in modulating artery reactivity (Lidington et al., 2022; Dinh et al., 2024). Thus, the absence of females in the present study limits its overall impact, since it cannot be presumed that females will have the same cardiac/vascular phenotype that has been observed in males. Other study limitations include: 1) visceral fat is known to influence blood pressure (Covassin et al., 2018), but an assessment of visceral *versus* subcutaneous fat content was not conducted; 2) we did not measure circulating factors, such as angiotensin II and endothelin-1, that are known to be elevated in obesity and have vascular effects (Barton et al., 2003); 3) our vascular assessments *ex vivo* were generally limited to myogenic reactivity and phenylephrine sensitivity; and 4) with the exception of radial arteries, we did not assess endothelial function. Including these additional measures would substantiate conclusions with regards to the modest *in vivo* phenotype and the lack of vascular phenotype *ex vivo*.

In summary, the present study shows that microvascular resistance artery function is preserved in this diet-induced model of obesity with metabolic dysfunction. Our study distinguishes this HFD model from other models of hypertension, which have a clear vascular component, and we conclude that resistance artery function is surprisingly resilient in a compromised metabolic environment.

## Data Availability

The original contributions presented in the study are included in the article/supplementary material, further inquiries can be directed to the corresponding author.
